# Food and nutrition literacy status and its correlates in Iranian senior high-school students

**DOI:** 10.1186/s40795-021-00426-2

**Published:** 2021-06-04

**Authors:** Marziyeh Ashoori, Nasrin Omidvar, Hassan Eini-Zinab, Elham Shakibazadeh, Azam Doustmohamadian, Behnaz Abdar-Esfahani, Mohammad Mazandaranian

**Affiliations:** 1 Student Research Committee, Department of Community Nutrition, National Nutrition and Food Technology Research Institute and Faculty of Nutrition Sciences and Food Technology, Shahid Beheshti University of Medical Sciences, Tehran, Iran; 2 Department of Community Nutrition, National Nutrition and Food Technology Research Institute (NNFTRI) and Faculty of Nutrition Sciences and Food Technology, Shahid Beheshti University of Medical Sciences, West Arghavan St. Farahzadi Blvd., Sharake Qods, Tehran, Iran; 3 Department of Health Education and Promotion, School of Public Health, Tehran University of Medical Sciences, Tehran, Iran; 4 Gastrointestinal and Liver Disease Research Center, Iran University of Medical Sciences, Tehran, Iran

**Keywords:** Food literacy, Iran, Nutrition literacy, Young adults

## Abstract

**Background:**

Planning interventions to promote food and nutrition literacy (FNL) require a better understanding of the FNL status of the target group and its correlates.

**Aims:**

This study aimed to examine the FNL status and its determinants in Iranian senior high-school students.

**Methods:**

In this cross-sectional study, FNL and its components (food and nutrition knowledge, functional skills, interactive skills, advocacy, critical analysis of information, and food label reading skill) were evaluated by a locally designed and validated, self-administered questionnaire. Besides, socioeconomic, demographic, anthropometric measures, as well as academic performance of 626 senior high-school students were assessed.

**Results:**

The mean ± SD of the total FNL score (within potential range of 0 to 100) was 52.1 ± 10.96, which is below the minimum adequate level of 60. The probability of high FNL knowledge score was significantly higher among students who majored in Natural Sciences (OR = 1.73, CI = 1.09–2.75), had better school performance (OR = 1.13, CI = 1.06–1.20) and higher SES score (OR = 1.20, CI = 1.01–1.44). The score for food label reading was significantly lower in girls (OR = 0.45, CI = 0.31–0.67), while those who had a family member with the nutrition-related disease were more likely to have a higher score of food label reading skill (OR = 1.48, CI = 1.01–1.64).

**Conclusion:**

The level of FNL in senior high-school students in Tehran was relatively low. These findings have key messages for the education system and curriculum designers to have more consideration for food and nutrition-related knowledge and skills in schools.

**Supplementary Information:**

The online version contains supplementary material available at 10.1186/s40795-021-00426-2.

## Introduction

Unhealthy eating behavior is among the leading risk factors of non-communicable diseases (NCDs) [1], which were responsible for 71% of global deaths in 2016 [2]. Based on an estimate, in 2017, one in every 5 deaths was preventable through improving dietary intake globally [3]. Therefore, poor dietary practice is one of the major concerns of the health sector in both developed and developing countries. In this regard, adolescents’ dietary behavior is particularly a concern, since poor dietary intakes are highly prevalent among this age group in both low- and middle-income countries [4]. In Iran, as a middle-income country, high consumption of fast foods and unhealthy snacks, skipping breakfast, and low intake of fruits, vegetables, whole grains, and dairy products have been reported as common inappropriate dietary practices among youth [5].

Previous studies has identified numerous determinants of diet quality, including demographics, socio-economic, environmental, and socio-cultural factors [6], as well as food and nutrition-related knowledge and skills [7]. Therefore, many nutrition education interventions have tried to promote healthy eating behavior(s); however, most of these interventions have had a traditional approach through focusing on nutrition knowledge rather than skills that have resulted in limited improvement in dietary intakes and/or practices [8].

Food literacy and nutrition literacy are the emerged concepts that address not only knowledge but skills about food and nutrition. Vidgen et al. have defined food literacy as the “collection of inter-related knowledge, skills and behaviors required to plan, manage, select, prepare and eat foods to meet needs and determine food intake” [9]. “Food literacy” and “nutrition literacy” are often used interchangeably [10]. Reviewing available literature indicates these concepts have considerable overlaps and complementarities, and aim at the same goal, i.e. promoting healthy and sustainable food choices. Therefore, it is hard to determine the definite border between “food literacy” and “nutrition literacy”. It seems that “food and nutrition literacy” (FNL) is a more comprehensive term to describe the set of knowledge and skills by which people can “plan, manage, select, prepare and eat foods” [9] and “make appropriate nutrition decisions” [10].

Late adolescents and youth are in a transition stage between adolescence and adulthood and are starting to experience or have recently experienced independent living. Consequently, they start to have more responsibilities in planning, selecting and, preparing foods compared to younger ages. Therefore, the knowledge and skill level of this age group about food and nutrition could help them cope with the complex and multifaceted factors influencing their dietary practice and this could have a critical impact on their eating habits and health in later life. In this regard, the high-school period can be considered as the last opportunity for the formal education system to improve the FNL level of adolescents and prepare them for future life. Despite this fact, nutrition education is not incorporated properly and adequately in school curriculums in many countries, including Iran. A recent content analysis of high school textbooks and curriculums with regard to FNL in Iran showed that there is very limited attention given to different components of FNL, specifically skill domain [11]. Thus, assessing the FNL status of late adolescence and youth could provide valuable evidence about a major need in this age group and possible gaps in the education system to be considered for future interventions and/or high school curriculum revisions.

Quantitative research in the field of food and nutrition literacy are limited but growing [12–21]. There are a number of studies on the FNL status of different age groups, including adults [12, 13, 17, 20, 21], youth [14, 19], adolescent [15, 18], and primary school children [16]. However, in many of these studies food or nutrition literacy is limited to nutrition knowledge [21], food label interpretation skill [13, 14], or both [12, 15, 20]. However, food and nutrition literacy as a multidimensional concept [22], encompasses much broader competencies, including food planning, shopping, budgeting, storage, and preparation skills, social aspects of eating, environmental sustainability, etc. [9]. Furthermore, no previous research has focused on FNL status of Iranian adolescents and youth and its components. Moreover, determinants of the FNL and its components in senior high-school students and youth are remained to be examined. Considering these gaps, the present study was carried out to examine the food and nutrition literacy status and its determinants in urban senior high-school students in Iran. The findings are expected to guide intervention planners in designing targeted and effective educational programs.

## Materials and methods

This cross-sectional study was carried out from November 2017 to April 2018 in the metropolitan city of Tehran.

### Study sample

The study participants were 755 senior high-school students (aged 17–18 years). Inclusion criteria included being enrolled in the senior high-school and willingness to participate. If a student was not interested to participate in the study or followed a special diet, he/she would be excluded from the study and were replaced by another student through random selection. The students were selected through the multistage cluster random sampling method. There are 19 educational districts in Tehran city which are classified into 3 socioeconomic levels, including: affluent (districts 1 to 6), semi-affluent (districts 7 to 14), and deprived (districts 15 to 19). Nine educational districts (3 from each socioeconomic level) were selected and the number of samples in each district, in private and public schools, from each sex and each study major (Natural Sciences**,** Mathematics, Literature, and Humanities) were determined according to the population proportion. The selection of educational districts and schools was performed through cluster random sampling and students were selected by stratified random sampling.

### Measures

#### Food and nutrition literacy

FNL was assessed by a 60-item Food and Nutrition Literacy Assessment Tool (FNLAT). The questionnaire was developed and validated for high-school graduates and youth in the prior stages of this project. The process of development of FNLAT and its validation has been reported elsewhere [23]. We used FNLAT, as the only valid multidimensional questionnaire available for Iranian adolescents and youth. This self-administered questionnaire comprised of two domains (knowledge and skills) and six dimensions (food and nutrition knowledge (27 items), functional skills (11 items), interactive skills (7 items), advocacy (7 items), critical analysis of information (5 items), and food label reading skill (3tiems)). FNLAT included 30 binary questions on nutrition knowledge and food label reading skills and 30 Likert-type statements for assessing other dimensions of skill domain. The domains and dimensions of FNLAT were developed based on domains and dimensions of FNL identified through the preliminary qualitative phase of the study. The area of knowledge and skills assessed in the questionnaire are listed in the Additional file 1. In addition, through construct validity assessment of the questionnaire, advocacy and critical analysis of information (the sub-dimensions of critical skills identified in qualitative phase), and food label reading skills items are included as two separate dimensions in FNLAT [23].

Total FNL score and each dimension’s score ranged from 0 to 100 (sum of the raw scores were linearly transformed to a score from 0 to 100), with higher scores indicating higher FNL level. In this questionnaire, FNL scores lower than 45 are interpreted as poor and those higher than 60 are considered as adequate food and nutrition literacy. Scores from 45 to 60 were categorized as moderate level of food and nutrition literacy. FNLAT was completed by the students while they were at school.

#### Anthropometric measurements

Participants’ weight was measured with minimum clothing, without shoes, using a digital scale (Seca) and recorded to the nearest 0.1 kg. Height was assessed using a wall-fixed tape in standing position, without shoes while shoulders were in a normal position. BMI for age z-score was calculated by using WHO Anthro-Plus software. Obesity and overweight were defined based on WHO criteria.

#### Socio-demographic variables and school performance

Socio-demographic variables were measured using a questionnaire which was completed through interview with students. In order to evaluate socioeconomic status (SES), participants were asked about their family size, household head, parents age, education and job position; ownership (or type in some cases) of home appliances and facilities (TV, washing machine, dishwasher, refrigerator, microwave, number of cars in the family, number of computers or laptops), and residential house features (house area, number of rooms and house ownership status). If needed, complementary phone interviews were performed with parents.

Also, academic performance was assessed using grade point average (GPA) earned in the national final exams which are taken at the end of the high school.

#### Statistical analysis

Descriptive statistics of demographic and anthropometric variables were reported by frequencies and percentages of distribution for categorical variables and mean ± SD for quantitative variables. Since several variables were measured as proxies of socioeconomic status (SES); in order to avoid multicollinearity in the regression model, principal component analysis (PCA) was applied to reduce socioeconomic variables into a unidimensional SES variable. The chi-square test was used to examine whether the distribution of categorical variables between boys and girls was significantly different. Student’s t-test and One-Way ANOVA were applied to compare means and variances of quantitative variables with normal distribution; scores of total FNL, its domains, and dimensions; by sex and other categorical variables. Pearson and Spearman correlation coefficient was calculated to examine the bivariate association between FNL with continuous and ordinal variables, respectively. In order to identify predictors of high FNL, logistic regression was applied, where poor food and nutrition literacy (score < 45) was considered as the reference group and adequate and moderate literacy were merged and considered as high FNL. The variables included in regression models as the possible predictors of FNL were: gender, study major, academic performance, SES score, presence of a nutrition-related disease in the participants or their family, and BMI-for-age z-score. The statistical significance level was set at *p* <  0.05. SPSS 21.0 (SPSS Inc., Chicago, Illinois, USA) software was used to perform all statistical analysis.

## Results

Of 755 students randomly selected, 621 provided all demographic and FNL data (response rate = 82.2%). Among the students with completed demographic data (*n* = 626), 49.7% (*n* = 311) were girls and 50.3% (*n* = 315) were boys. A comparison between subjects who included in the analysis and excluded ones showed no significant difference in socio-demographic characteristics (parent education and job position, city district) (*p* > 0.05) except for gender. Of excluded subject, 58.2% were girls and 41.8% were boys (*p* = 0.04).

Demographic and anthropometric characteristics of the study participant are shown in Table 1. The mean age of students was 17.82 ± 0.39 years. Education level in most of the parents was high school diploma or lower (74.2% of mothers and 68.4% of fathers). There were no significant differences between boys’ and girls’ general characteristics, except for study major (*p* <  0.001), weight status (*p* <  0.01), academic performance (p <  0.001), and presence of nutrition-related diseases (in the students or their family) (p <  0.001) (Table 1).

**Table 1:** Demographic and anthropometric characteristics of study participants

	n (%) or Mean ± SD			
	Total	Girls	Boys	*P* value
**City district**				0.472^a^
High SES district	327 (52.2)	168 (54.0)	159 (50.5)	
Middle SES district	154 (24.6)	70 (22.5)	84 (26.7)	
Low SES district	145 (23.2)	73 (23.5)	72 (22.9)	
**School type**				0.459^a^
Public	441 (70.4)	218 (70.1)	223 (70.8)	
Private	185 (29.6)	93 (29.9)	92 (29.2)	
**Major**				< 0.001 ^a^
Literature and Humanities	144 (23.0)	89 (28.6)	55 (17.5)	
Natural Sciences	215 (34.3)	127 (40.8)	88 (27.9)	
Mathematics	267 (42.7)	95 (30.5)	172 (54.6)	
**Father education**				0.389^a^
Illiterate	11 (1.8)	7 (2.3)	4 (1.3)	
Less than high-school diploma	127 (20.3)	56 (20.9)	62 (19.7)	
High-school diploma	290 (46.3)	152 (48.9)	138 (43.8)	
Associate degree or bachelor	136 (21.7)	60 (19.3)	76 (24.1)	
MSc or PhD	61 (9.7)	27 (8.7)	34 (10.8)	
Dead	1 (0.2)	0	1 (0.3)	
**Mother education**				0.492^a^
Illiterate	15 (2.4)	7 (2.3)	8 (2.5)	
Less than high-school diploma	130 (20.8)	72 (23.2)	58 (18.4)	
High-school diploma	319 (51.0)	159 (51.1)	160 (50.8)	
Associate degree or bachelor	138 (22.0)	61 (19.6)	77 (24.4)	
MSc or PhD	24 (3.8)	12 (3.9)	12 (3.8)	
**Father job position**^c^				0.389^a^
Unemployed	9 (1.4)	4 (1.3)	5 (1.6)	
Worker	77 (12.3)	40 (12.9)	37 (117)	
Clerk	151 (24.1)	69 (22.2)	82 (26.0)	
Self-employed jobs	232 (37.1)	114 (36.7)	118 (37.5)	
High income jobs	69 (11.0)	43 (13.8)	26 (8.3)	
Retired	73 (11.7)	33 (10.6)	40 (12.7)	
Dead or divorced	15 (2.4)	8 (2.6)	7 (2.2)	
**Mother job position**^c^				0.193^a^
Unemployed	522 (83.4)	261 (83.9)	261 (82.9)	
Worker	8 (1.3)	6 (1.9)	2 (0.6)	
Clerk	68 (10.9)	27 (8.7)	41 (13.0)	
Self-employed jobs	14 (2.2)	7 (2.3)	7 (2.2)	
High income jobs	6 (1.0)	5 (1.6)	1 (0.3)	
Retired	7 (1.1)	4 (1.3)	3 (1.0)	
Dead or divorced	1 (0.2)	1 (3.0)	0	
**Weight status** (based on BMI-for-age Z-scores)				0.009^a^
Normal weight (z-scores <1SD)	337 (56.4)	177 (61)	160 (51.9)	
Overweight (1SD ≤ z-scores <2SD)	147 (24.6)	71 (24.8)	75 (24.4)	
Obese (z-scores ≥2SD)	114 (19.1)	41 (14.1)	73 (23.7)	
**Nutrition related disease**^d^				< 0.001^a^
Don’t have	259 (83.3)	292 (92.7)	551 (88)	
Have	52 (16.7)	23 (7.3)	75 (12)	
**Nutrition related disease in family**^d^				< 0.001^a^
Don’t have	187 (60.1)	244 (77.5)	431 (68.8)	
Have	124 (39.9)	71 (22.5)	195 (31.2)	
**Academic performance (GPA)**^e^	14.90 ± 2.64	13.1 ± 3.16	14.00 ± 3.05	< 0.001^b^
**SES score (factor score)**^f^	−0.017 ± 0.98	0.016 ± 1.01	0.00 ± 1.00	0.67^b^
**Student age (Yr.)**	17.82 ± 0.39	17.79 ± 0.37	17.85 ± 0.41	0.093^b^
**Father age (Yr.)**	48.62 ± 5.54	48.49 ± 5.46	48.75 ± 5.62	0.563^b^
**Mother age (Yr.)**	43.18 ± 5.25	43.48 ± 5.25	42.89 ± 5.24	0.163^b^

^a^ Chi-squared test

^b^ Student’s t-test

^c^Workers are defined as people who may not have a permanent jobt and income (low-income job); Clerks are those who employed in an office or company with a consistent income (relatively low income); self-employed jobs such as shopkeepers, barbers, car mechanics, etc.; high-income jobs included factory owner, Jewelry storeowner, etc.

^d^Nutrition related disease; suffering from diabetes, hypertension, dyslipidemia, cardiovascular diseases and cancer

^e^GPA: grade point average (within potential range of 0 to 20)

^f^SES has been calculated using principal component analysis (the factor score saved as the SES variable)

### Food and nutrition literacy status and its correlates

**Fig. 1:**
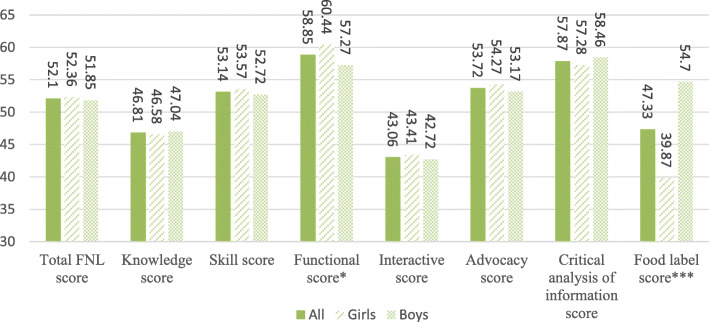
Mean scores of food and nutrition literacy and its domains and dimensions. * Mean score is significantly different between boys and girls at *p* < 0.05, using student’s t-test. *** Mean score is significantly different between boys and girls at *p* < 0.001, using student’s t-test

Mean total FNL score and its domains and dimensions are shown in Fig. 1. The mean ± SD of the total FNL score was 52.1 ± 10.96 with no significant difference between boys and girls. Girls had significantly higher functional score than boys, while food label score was significantly higher in boys as compared to girls. Among the FNL dimension, the highest score belonged to functional skills (58.85 ± 18.66), while the mean score of interactive skills was the lowest (43.06 ± 18.40).

The results of the bivariate analysis of FNL determinant factors are presented in Tables 2 and 3. As shown in Table 2, a significant positive but poor correlation was found between knowledge score and academic performance (r = 0.188), mother education (r = 0.113), and SES score (r = 0.153). Higher BMI-for-age Z-score was also significantly correlated with functional scores (r = 0.115). The correlation between BMI-for-age Z-score and total FNL, knowledge, and skill score was statistically significant but negligible (r <  0.1). Father education and job position had also a significant but negligible correlation with knowledge (r = 0.084) and critical analysis of information score (r = 0.084), respectively. As presented in Table 3, the mean knowledge score was significantly lower in students who studied Literature and Humanities compared to those who majored in Natural Sciences and Mathematics; and in students of public versus private schools (*p* <  0.05). Critical analysis of information scores was significantly higher in students of public schools compared to those in private schools and in students whose major was mathematics compared to those who studied Natural sciences and in students who suffered from a nutrition-related disease compared to healthy ones (p <  0.05). Regarding food label reading skill, participants who studied mathematics had a significantly higher mean score than those whose major was literature and humanity (*p* < 0.05).

**Table 2:** Correlation coefficient between food and nutrition literacy, its domains and dimensions and some possible determinant factors

	Correlation coefficient							
	Total FNL and its domains			Skill dimensions				
	Total FNL	Knowledge score	Skill score	Functional score	Interactive score	Critical analysis of information score	Advocacy score	Food label reading score
Academic performances ^a^	0.023	0.188^***^	- 0.034	- 0.029	- 0.070	- 0.001	- 0.008	- 0.004
Mother education ^b^	0.024	0.113^**^	- 0.005	0.032	- 0.009	0.050	- 0.011	0.041
Father education ^b^	0.048	**0.084** ^*^	0.031	0.036	0.023	0.047	- 0.024	0.074
Mother job position ^b^	0.002	0.074	- 0.016	0.008	- 0.006	- 0.060	- 0.041	0.027
Father job position ^b^	0.041	0.066	0.028	0.016	0.001	0.084^*^	- 0.004	- 0. 047
BMI for age Z-score ^a^	**0.098** ^*^	0.091^*^	0.084^*^	**0.115** ^*^	0.044	0.014	- 0.013	0.057
SES score ^a^	0.065	0.153^***^	0.029	0.056	0.029	0.021	- 0.071	0.047

^a^ values are Pearson correlation coefficient

^b^ values are Spearman correlation coefficient

^*^ *P* < 0.05

^**^ *P* < 0.01

^***^ *P* < 0.001

**Table 3:** Mean score of food and nutrition literacy, its domains and dimensions by categories of some possible determinant factors

	Mean ± SD							
	Total FNL and its domains			Skill dimensions				
	Total FNL	Knowledge score	Skill score	Functional score	Interactive score	Critical analysis of information score	Advocacy score	Food label reading score
Major ^*^								
Literature and Humanities	51.88 ± 11.5	**42.79 ± 15.3**^**a**^	53.99 ± 12.3	60.37 ± 18.7	45.28 ± 19.5	56.87 ± 15.0	54.41 ± 19.8	**40.50 ± 31.8**^**a**^
Natural Sciences	51.82 ± 10.2	**48.97 ± 12.7**^**b**^	53.44 ± 11.1	59.63 ± 17.6	43.23 ± 17.7	**55.99 ± 16.2**^**a**^	55.06 ± 20.4	44.74 ± 33.9
Mathematics	51.64 ± 11.1	**47.24 ± 15.4**^**b**^	52.45 ± 12.1	57.40 ± 19.3	41.72 ± 18.1	**59.92 ± 16.7**^**b**^	52.27 ± 20.1	**50.93 ± 32.47**^**b**^
School type^†^								
public	51.99 ± 11.1	**45.83 ± 14.4**^**a**^	53.23 ± 12.1	58.81 ± 18.2	43.44 ± 18.6	**56.99 ± 15.3**^**a**^	54.64 ± 20.1	47.69 ± 31.6
private	52.38 ± 10.4	**49.14 ± 15.1**^**b**^	52.94 ± 11.1	58.95 ± 19.5	42.15 ± 17.7	**60.00 ± 18.1**^**b**^	51.49 ± 20.1	46.48 ± 36.2
Nutrition related disease^†^								
Don’t have	51.95 ± 11.0	46.58 ± 14.6	52.98 ± 11.9	58.36 ± 18.6	43.32 ± 18.4	**57.39 ± 16.1**^**a**^	53.73 ± 20.2	48.03 ± 32.8
Have	53.44 ± 10.5	48.49 ± 15.1	54.34 ± 11.1	62.44 ± 18.7	41.11 ± 17.7	**61.44 ± 16.8**^**b**^	53.60 ± 20.1	42.22 ± 34.3
Nutrition related disease in family^†^								
Don’t have	51.87 ± 10.9	46.63 ± 14.8	52.91 ± 11.8	58.25 ± 18.1	42.77 ± 18.7	58.36 ± 15.99	53.51 ± 20.2	47.09 ± 33.0
Have	52.61 ± 11.0	47.21 ± 14.4	53.65 ± 11.8	60.16 ± 19.8	43.69 ± 17.7	56.80 ± 16.8	54.17 ± 20.2	47.86 ± 33.1

Nutrition related disease; suffering from diabetes, hypertension, dyslipidemia, cardiovascular diseases and cancer

^a, b^ Values with different superscript are significantly different (*p* < 0.05)

^*^Statistical significance of means difference was examined using One-Way ANOVA

^†^Statistical significance of means difference was examined using Student’s t-test

Possible socio-demographic predictors of FNL and its domains and dimensions were examined through multivariate analysis (Table 4). The probability of high knowledge score was significantly higher among students who studied Natural Sciences compared to those whose major were Literature and Humanities (OR = 1.73, CI = 1.09–2.75). Higher SES score (OR = 1.20, CI = 1.01–1.44) and better academic performance (OR = 1.13, CI = 1.06–1.20) were also associated with increased probability of having higher knowledge score (OR = 1.13, CI = 1.06–1.20). Better academic performance was associated with lower probability of high functional (OR = 0.93, CI = 0.87–0.99) and interactive (OR = 0.92, CI = 0.87–0.98) score. In female students, the probability of high food label reading skill was 55% less than male students (*p* < 0.001). Having a nutrition related-disease in the family members increased the probability of higher food label reading skill by 48% (*p* < 0.05).

**Table 4:** Factor associated with higher FNL, its domains and dimensions

	Odds ratio (0.95% confidence interval)^a^							
	Total FNL and its domains			Skill dimensions				
	High total FNL	High knowledge score	High skill score	High functional score	High interactive score	High critical analysis of information score	High advocacy score	High Food label reading score
gender								
boy	Reference	Reference	Reference	Reference	Reference	Reference	Reference	Reference
girl	1.17 (0.76–1.82)	0.78 (0.53–1.13)	1.29 (0.83–2.09)	1.33 (0.86–2.05)	1.19 (0.82–1.72)	1.07 (0.67–1.70)	1.19 (0.81–1.76)	**0.45 (0.31–0.67)** ^***^
Major								
Literature and Humanities	Reference	Reference	Reference	Reference	Reference	Reference	Reference	Reference
Natural Sciences	1.411 (0.81–2.43)	1.73 (1.09–2.75)^*^	1.16 (0.67–2.00)	1.10 (0.63–1.13)	0.92 (0.58–1.44)	0.70 (0.39–1.24)	0.98 (0.60–1.59)	1.33 (0.84–2.10)
Mathematics	0.80 (0.48–1.34)	1.39 (0.88–2.20)	0.91 (0.53–1.54)	0.82 (0.48–1.40)	0.82 (0.52–1.29)	0.95 (0.53–1.72)	0.87 (0.54–1.61)	1.51 (0.95–1.2.38)
Academic performance	1.02 (0.96–1.10)	1.13 (1.06–1.20)^***^	0.98 (0.92–1.05)	**0.93 (0.87–0.99)** ^*^	**0.92 (0.87–0.98)** ^*^	0.98 (0.91–1.06)	1.02 (0.96–1.08)	1.03 (0.97–1.09)
SES score	1.20 (0.98–1.47)	1.20 (1.01–1.44)^*^	0.97 (0.79–1.19)	1.07 (0.87–1.31)	1.02 (0.86–1.22)	1.06 (0.85–1.32)	0.85 (0.71–1.02)	0.99 (0.83–1.18)
Nutrition-related disease								
Don’t have	Reference	Reference	Reference	Reference	Reference	Reference	Reference	Reference
Have	1.05 (0.56–1.96)	0.83 (0.49–1.43)	1.14 (0.62–2.09)	0.71 (0.36–1.40)	1.71 (0.99–2.95)	0.67 (0.32–1.38)	1.04 (0.59–1.81)	1.29 (0.75–2.2)
Nutrition-related disease in family								
Don’t have	Reference	Reference	Reference	Reference	Reference	Reference	Reference	Reference
Have	0.86 (0.56–1.96)	1.07 (0.73–1.56)	0.82 (0.53–1.27)	0.98 (0.63–1.52)	1.28 (0.88–1.85)	0.83 (0.52–1.32)	0.96 (0.65–1.41)	**1.48 (1.01–1.64)** ^*^
BMI for age	1.11 (0.96–1.29)	1.09 (0.96–1.23)	1.07 (0.92–1.23)	1.06 (0.91–1.22)	1.03 (0.911–1.16)	1.03 (0.88–1.20)	1.01 (0.89–1.15)	1.09 (0.96–1.24)

*FNL* food and nutrition literacy. Nutrition-related disease; suffering from diabetes, hypertension, dyslipidemia, cardiovascular diseases and cancer

^a^Poor food and nutrition literacy (score < 45) was considered as reference group, adequate and moderate literacy were merged and considered as high FNL

^*^ *P* < 0.05

^***^ *P* < 0.001

## Discussion

The findings of the present study showed that mean score in none of the FNL domains and dimensions was above the adequate level (≥60) which indicates that the FNL status of Iranian youth needs improvement. Considering the fact that the study participants were senior high-school students who had completed formal education, their FNL status conveys key messages for the educational system and could reflect the weakness points of current school curricula in improving food and nutrition literacy among students.

There were no significant differences between boys and girls scores in overall FNL and its dimensions, except for functional and food label reading skill scores. The mean score of functional skills was slightly higher in girls compared to boys; however, after adjusting for other factors in the multivariate analysis, gender was not a significant predictor of functional skills anymore. On the other hand, with regard to food label reading skill, gender was a strong predictor even after adjusting the effect of all other possible predictors in multivariate analysis. The results showed that boys scored higher in reading and interpreting food labels. Reviewing the available literature indicate that there is no consistent gender difference in food label use or interpreting skills. Some studies did not show any gender differences [24–26], while some indicated that females more frequently used or correctly interpreted food labels compared to males [27–29]. According to the literature, women seem to use food labels more frequently than men [25, 27, 28]; however, inconsistency between studies exist regarding interpreting and understanding food labels [24–26, 29], suggesting that other factors such as age, education level, nutrition knowledge, etc., may affect gender differences.

Based on bivariate analysis, mother education level, studying in private school and higher SES score were significantly associated with higher food and nutrition knowledge score. Multivariate analysis confirmed these results; as increasing SES score was associated with a higher likelihood of higher knowledge score. Consistent results have been reported in several studies [12, 30–33]. Aihara et al. indicated that higher educational level and economic status was associated with adequate nutrition literacy in elderly Japanese [30]. Although they used the term “nutrition literacy”, but their questionnaire only assessed nutrition knowledge. Similarly, other studies have shown higher education level [12, 31–33] and job position [12, 31, 32] were positively associated with nutrition knowledge. The necessity of food and nutrition knowledge as a prerequisite for dietary changes [8], although not sufficient, calls the need for more emphasis on nutrition educational programs targeted at lower SES groups.

Academic performance was also associated with higher knowledge score, but surprisingly with lower functional and interactive scores. This may be due to the fact that current high school curriculums and textbooks in the country have relatively little on food and nutrition which is dominantly focused on knowledge aspects [11]. Besides, students with better academic performance due to heavy school workload, may have limited time or interest to develop their food and nutrition-related skills i.e. food shopping, preparation, and cooking (functional skills) or interact with others about food and nutrition (interactive skills). This may be especially more relevant in our study participants who were senior high-school students preparing for the university entrance exam. Further research is needed to make a more reliable conclusion in this regard.

The possibility of higher knowledge score was significantly higher in students who studied Natural Sciences compared to those whose major were Literature and Humanities. Food and nutrition-related topics are more likely to be discussed in the Natural Sciences courses than other majors. A recent analysis of the content of high school textbooks in Iran showed that food and nutrition-related topics have been addressed more frequently in textbooks of Natural Sciences than other majors [11] which confirmed the results of the current study.

The weight and health status of the subjects were also examined as possible determinants of FNL and its dimensions. Higher BMI was correlated with higher functional score in bivariate analysis. However, after controlling for the effect of other possible predictors in multivariate analysis, this association was not significant anymore. The relationship between weight status and FNL has been addressed in a number of studies [12, 15, 20, 30, 34, 35]; however, the results have not been consistent. In some studies, people with higher BMI have had a lower level of FNL [20, 34], while in some others non-significant [12, 15, 30] or positive [35] association between BMI and FNL has been reported. These investigations have been conducted among different age and sex groups that may partly explain this inconsistency in findings. In a study conducted by Kubiet et al. among adolescents [15], multivariate analysis showed no significant association between weight status and FNL, which is consistent with our findings. However, the limited number of studies, all with cross-sectional design makes it hard to make a conclusion.

In the current study, the presence of nutrition-related diseases in a family member predicted the possibility of higher food label reading skill of the students. Previous reports have also been indicated that people who suffer from nutrition-related diseases e.g. hypertension, diabetes, cardiovascular disease, etc., pay more attention to food labels [36]. People with nutrition-related chronic diseases and their families have more concerns about diet and may want to limit the consumption of some specific dietary components like calories, sugar, fat, salt, etc. These concerns could explain higher food label interpreting skills among people with chronic diseases and their families.

To the best of our knowledge, this is the first study assessing the FNL status of Iranian senior high-school students by a valid multidimensional tool. However, this study had some limitations that need to be taken into consideration. First, its cross-sectional design makes it impossible to interpret the direction of the associations. Moreover, the determinant factors examined in the current study could not explain the variation in score of the skill domain and its dimensions well. It seems that more complex factors affect FNL related skills which had not been included in our study. For example, food skills may be affected by socio-cultural norms which were not assessed in the present study. Therefore, in order to explore possible determinant factors of the FNL skill domain, further research especially with qualitative design could provide more insights. Finally, this study conducted among senior high-school students in Tehran; therefore, its results may not be generalized to other age groups or different populations.

In conclusion, the present study showed that Iranian senior high-school students have relatively low food and nutrition-related knowledge and skills. Among possible determinant factors examined, study major, academic performance and SES were significant predictors of youth’s food and nutrition knowledge; and male gender and having nutrition-related diseases in family members were determinant factors of higher food label reading skill. Further studies are recommended to identify other possible factors related to youth’s FNL. The findings reemphasize the need for evaluating current formal education curriculums with regard to food and nutrition knowledge and skill development as an important competent of life skills. Also, relatively low FNL level among senior high-school students highlighted the need for future studies focusing on FNL promoting interventions in high school students in Iran.

## Supplementary Information

**Additional file 1.** Identified domains, dimensions and sub-dimensions of Food and Nutrition Literacy for Iranian youth.

## Data Availability

The datasets used and/or analysed during the current study are available from the corresponding author on reasonable request.

## Abbreviations

NCDs: Non-communicable diseases
FNL: Food and nutrition literacy
FNLAT: Food and Nutrition Literacy Assessment Tool
GPA: Grade point average
