# Supplementary material for: Food and nutrition literacy status and its correlates in Iranian senior high-school students
Source: BMC Nutr. 2021 Jun 4;7:19. doi: 10.1186/s40795-021-00426-2 (PMC8176697; doi:10.1186/s40795-021-00426-2)
Supplement: Supplementary file 1 — Additional file 1. Identified domains, dimensions and sub-dimensions of Food and Nutrition Literacy for Iranian youth. [file 40795_2021_426_MOESM1_ESM.docx]

**Additional file 1**. Identified domains, dimensions and sub-dimensions of Food and Nutrition Literacy for Iranian youth.

| Domains | Dimensions | Sub-dimensions |
| --- | --- | --- |
| Knowledge | Food and nutrition knowledge | Knowledge of nutrition basic  Knowledge of shopping, storage and preparation of foods  Knowledge of food production and environmental sustainability  Cultural and social issues related to food |
| Skills | Functional skills | Applying basic food and nutrition knowledge  Shopping and storage of foods and meal preparation skills  Food production and environmental sustainability related skills |
|  | Interactive skills | Seeking food and nutrition information  Interact with others (family, friends, practitioners, etc.) about food and nutrition |
|  | Critical skills | Analysis of food and nutrition information critically  Advocacy for promoting healthy and sustainable food choice |
